# Linear Nevus Sebaceous Syndrome: Clinical Presentation and Management Considerations

**DOI:** 10.7759/cureus.60487

**Published:** 2024-05-17

**Authors:** Meghavi Pandya, Archana R Thool, Sachin Daigavane

**Affiliations:** 1 Department of Ophthalmology, Jawaharlal Nehru Medical College, Datta Meghe Institute of Higher Education and Research, Wardha, IND

**Keywords:** vascular conjunctival lesion, choristomas, optic disc coloboma, linear nevus sebaceous syndrome, neurocutaneous condition

## Abstract

A relatively rare neurocutaneous condition known as linear nevus sebaceous syndrome (LNSS) is marked by a variety of symptoms as well as the unusual characteristics of developing mosaic RASopathies of phacomatoses. Mental retardation, seizures, and midline facial linear nevus sebaceous were the usual diagnostic triad. A hallmark of LNSS is the papillomatous growth of the epidermis, also known as nevus sebaceous. In this case report, we presented a case of a 13-year-old male with LNSS with a vascular conjunctival lesion and a coloboma of the optic disc.

## Introduction

Linear nevus sebaceous syndrome (LNSS) is a rare neurocutaneous illness that is characterized by a wide range of symptoms and peculiar features of developing mosaic RASopathies of phacomatoses [1-3]. A typical triad for diagnosis included mental retardation, seizures, and midline face linear nevus sebaceous. The papillomatous proliferation of the epidermis, or nevus sebaceous, is a characteristic of LNSS [4]. Schimmelpenning documented LNSS for the first time in 1957 [5]. The midline facial linear nevus sebaceous, neurologic abnormalities, and ophthalmologic abnormalities should be added to the triad for this condition, according to Lambert et al.'s (1987) proposal. They redefined the disease as an oculo-neuro-cutaneous syndrome. A different term for this association is organoid nevus syndrome. Ocular involvement can occur in up to 60% of cases. Although the clinical presentations vary, strabismus, colobomas, and choristomas are the most prevalent abnormalities. The syndrome's most defining ocular trait is complex choristoma, yet it is not its unique feature. Iris and chorioretinal colobomas, asymmetry of orbital bones, antimongoloid lid fissures, generalized retinal degeneration, restricted choroidal hemangioma, and other uncommon disorders are also listed [6,7]. In addition to ocular characteristics, the broad spectrum of anomalies in LNSS can affect multiple organ systems, including the central nervous system (which includes brain tumors, lateral ventricle enlargement, and hemimegalencephaly). Seizures account for 75% of neurological manifestations, while intellectual deficits make up 60%. In this case report, we present a case of LNSS with a vascular conjunctival lesion and a coloboma of the optic disc.

## Case presentation

**Figure 1:**
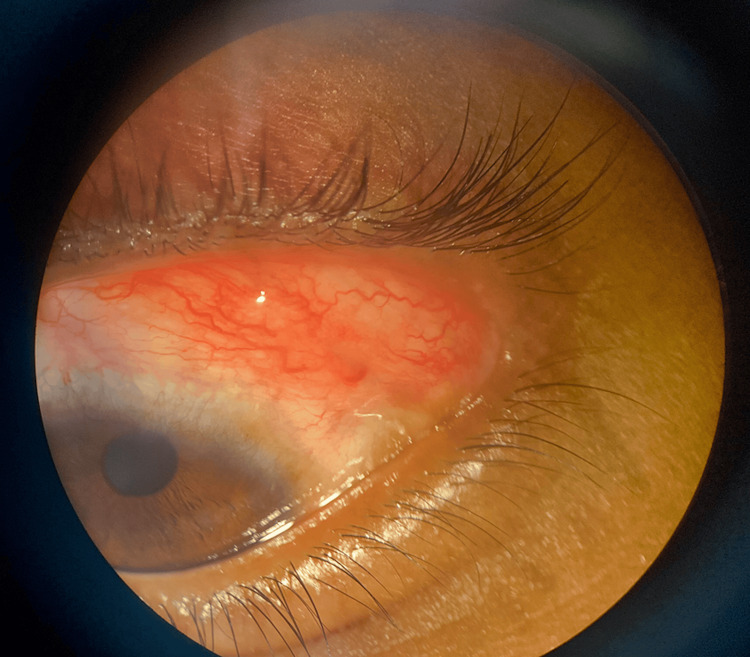
Vascular conjunctival lesion present over the superolateral aspect of the left eye

**Figure 2:**
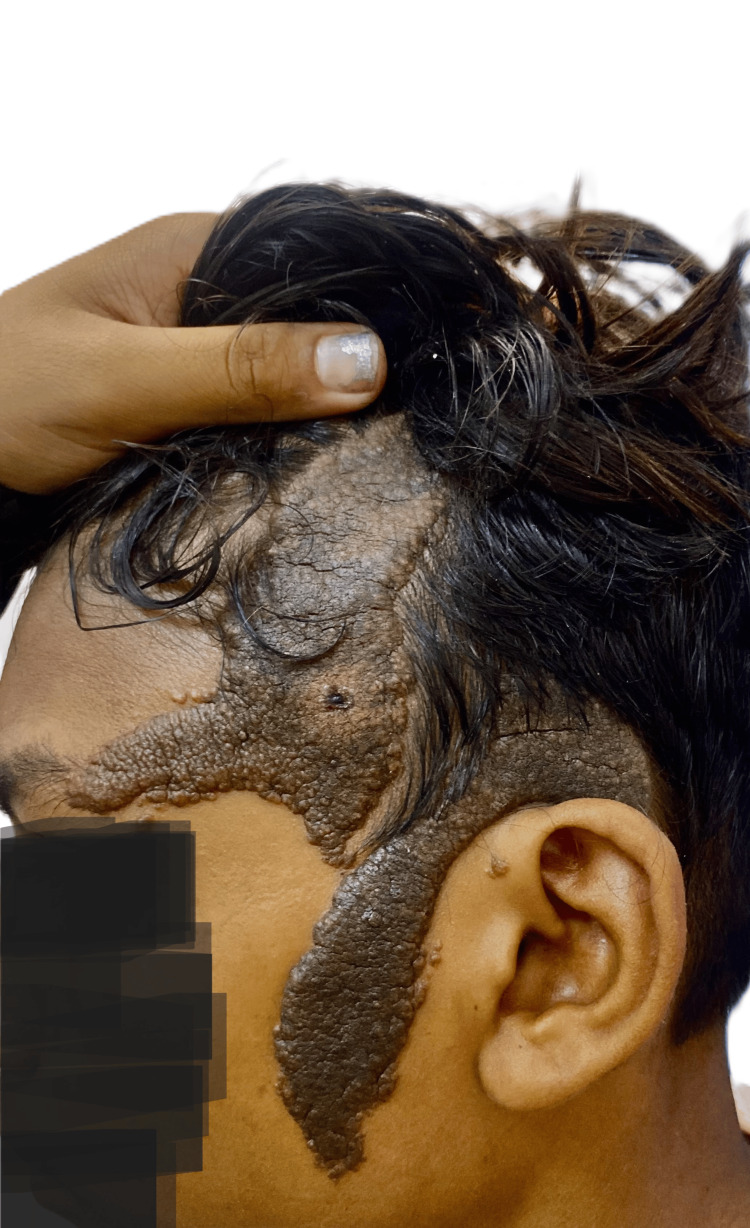
Linearly arranged, slightly elevated, hyperpigmented plaques over the left side of the forehead and face

A 13-year-old boy was brought to the outpatient ophthalmology department at Acharya Vinoba Bhave Rural Hospital, which is a part of Jawaharlal Nehru Medical College in Sawangi (Meghe) Wardha, India, with complaints of a reddish mass in the left eye since childhood and a dark-colored lesion over the left side of the head and cheek. As narrated by the father, the patient had a vascular mass in the left eye since birth, which was progressive and painless in nature, along with a nevus on the left paratemporal region and over the left cheek, gradually increasing in size and area. It was asymmetrical, white-colored, and changed to a black nevus with progression. Family history was evident of a consanguineous marriage. The patient was born at 40 weeks via normal vaginal delivery at home. All developmental milestones were achieved as per age, and no developmental delay was observed. The patient has been immunized up to age as per the National Immunization Surveys (NIS). He had a history of seizures since he was 5 years of age; the frequency of seizures was less than one per year, and the last episode was noted to be at 9 years of age. Upon ocular examination, he had a vascular conjunctival lesion of approximately 2.4 x 2 cm in size present over the superolateral aspect of the left eye (Figure 1). The lesion was painless and progressive in nature and did not obstruct the vision of the patient. The best corrected visual acuity (BCVA) was recorded to be 6/6 for both eyes. Intraocular pressure was recorded by non-contact tonometry and noted to be within normal limits for both eyes. The lesion involving the left side of the forehead and face, on examination, appeared to be a typical sebaceous nevus. These lesions were linearly arranged, slightly elevated, hyperpigmented plaques (Figure 2).

**Figure 3:**
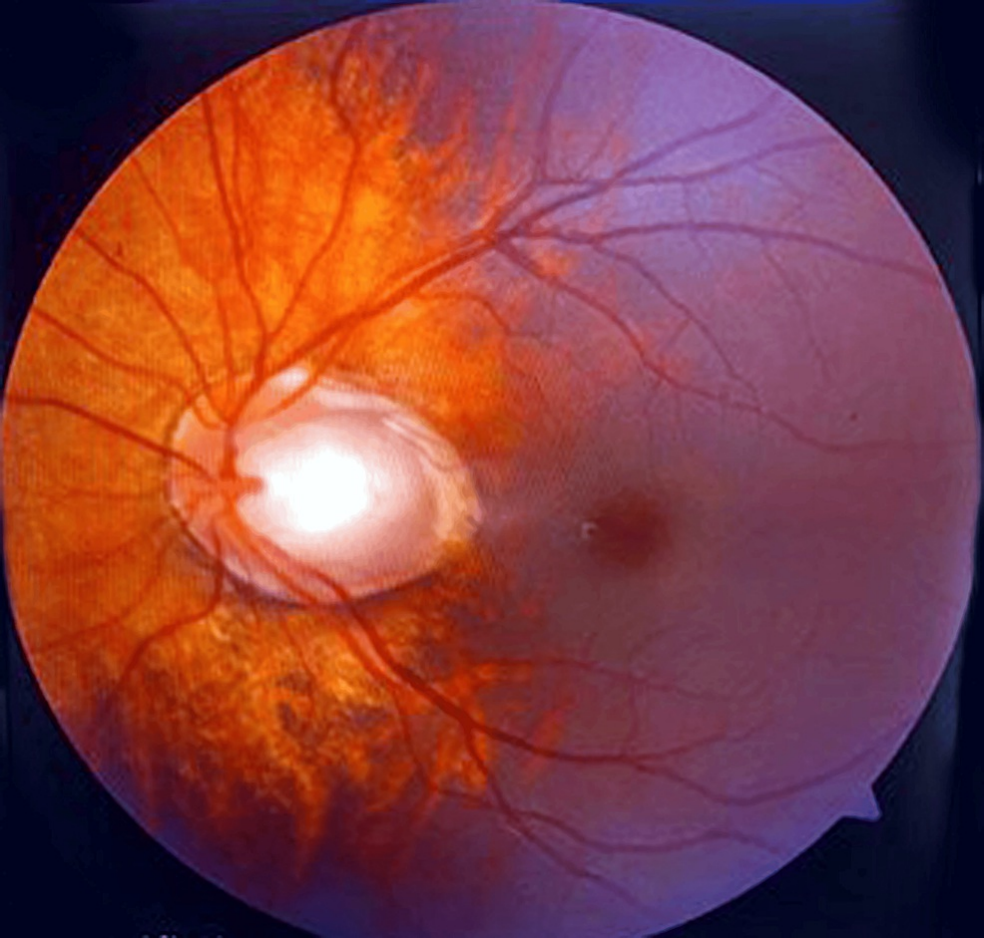
Left eye fundus image showing optic disc coloboma

The fundus examination of the left eye revealed a coloboma involving the optic disc with tessellations, a normal arteriovenous ratio of 2:3, and the presence of a foveal reflex (Figure 3). The right eye fundus was observed to be within the normal limits.

**Figure 4:**
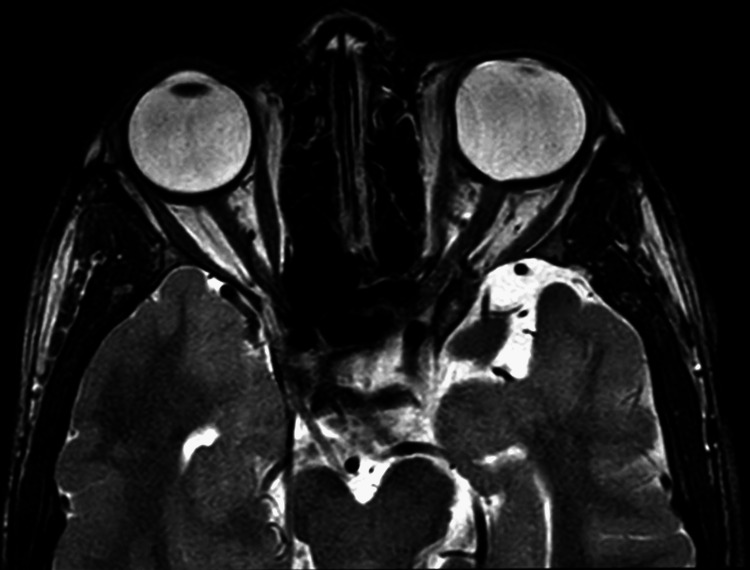
MRI-axial section T2WI of bilateral orbit showing a mild thickening of the optic nerves

**Figure 5:**
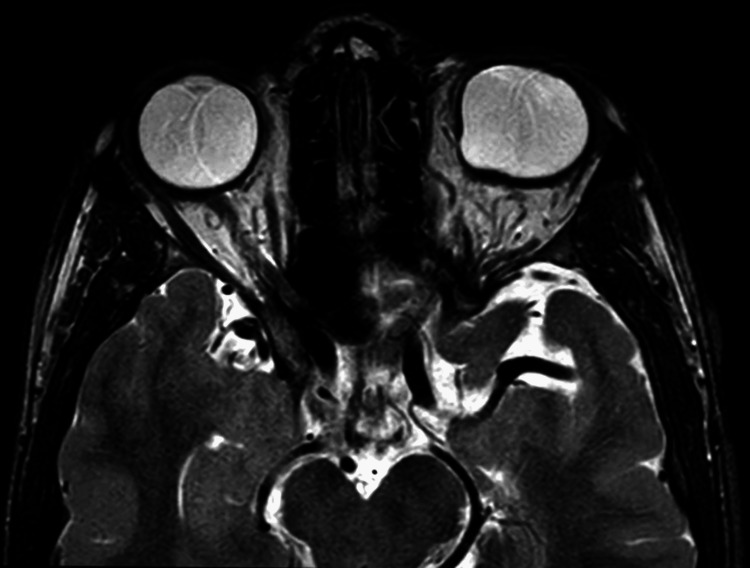
MRI-axial section T2WI of bilateral orbit suggestive of posterior staphyloma in the left globe

A magnetic resonance imaging (MRI) scan of the orbit (plain) was performed to investigate the conjunctival vascular lesion present in the superolateral aspect of the left eye in order to evaluate the posterior extension and boundaries of avascular conjunctival lesion present over the superolateral aspect of the left eye. On the plain MRI orbit, it was observed that the left globe is mildly bulky with an irregular scleral outline along the medial and posterior aspects with a mild protrusion along the posterior aspect; mild adjacent thickening of the optic nerves is seen; the right globe is in a normal shape, and the uveoscleral thickness is normal; the lens is in a normal position; the lacrimal glands appear normal; the extraocular muscles show normal thickness and shape on both sides; the optic nerves on both sides are normal in thickness; the retro bulbar fat is normal on both sides; the orbital apex, superior ophthalmic fissure, inferior ophthalmic fissure, and cavernous sinuses are normal; the sellar and parasellar regions and the optic chiasm appear normal; and the region of lacrimal sac and preseptal compartment appear normal. Mild adjacent thickening of the optic nerves is observed, which is suggestive of a left posterior staphyloma (Figures 4, 5).

 

**Figure 6:**
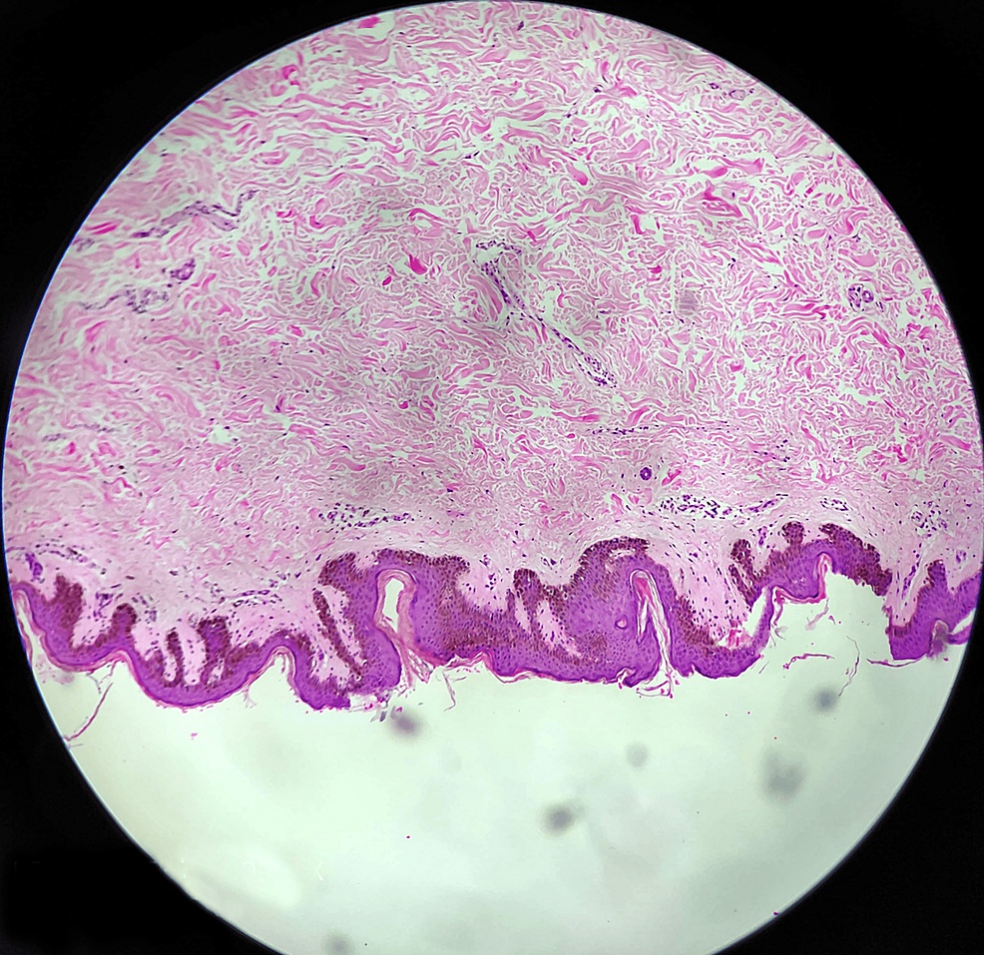
The sections from the provided tissue specimen show acanthosis, papillomatosis, and mild hyperkeratosis

Symptomatic treatment is applied with an emphasis on the particular organ affected. The patient was planned for an excision and split skin graft for the linear hyperpigmented plaque present on the left side of the forehead and face. A split-thickness skin graft was harvested from the right thigh, and the nevus tissue excision was performed. After achieving hemostasis, a meshed graft was placed, and suturing was done using Ethilon 3-0 and Vicryl 3-0. Subsequently, the excised tissue was sent for histopathological examination. A single cutaneous tissue specimen, with skin attached, measuring 20 x 6 x 0.2 cm, was provided for histopathological evaluation. The sections of the provided tissue specimen show acanthosis, papillomatosis, and mild hyperkeratosis. The histopathology reveals a focal absence of the granular layer underlying the dermis, with extensive areas of proliferation of sebaceous glands and primitive hair follicles. The histopathological features indicate the presence of nevus sebaceous (Figure 6).

The patient’s ocular manifestations did not obstruct his extraocular movements. The patient was given correction for refractive error, resulting in a BCVA of 6/6 for each eye. The patient was instructed to closely schedule a follow-up to evaluate the progression of the ocular lesion and determine whether surgical excision is unequivocally indicated.

## Discussion

In addition to its extracutaneous manifestations, LNSS is a neurocutaneous condition affecting the epidermis. The diagnosis of LNSS is typically made clinically, while some patients may require genetic testing in addition to a comprehensive neurological and ocular workup. We have highlighted a rare neurocutaneous condition in our case report that is typically linked to neurologic abnormalities, ophthalmologic abnormalities, and the midline face linear nevus sebaceous. The patient presents with a vascular conjunctival lesion across the superolateral aspect of the left eye, along with a typical sebaceous nevus encompassing the left part of the forehead and face. Upon examination, these lesions were found to be linearly arranged, slightly elevated, hyperpigmented plaques. During the fundus evaluation, a coloboma involving the optic disc was noted in the left eye. The patient’s MRI orbit report suggested left posterior staphyloma. There was an evident history of consanguineous marriage, and four to five episodes of seizures were noted, the last being reported at the age of 9 years. The patient had no restriction of eye movements and had a BCVA of 6/6 for both eyes. No signs of mental retardation were seen.

The relationship between the ocular symptoms of oculoectodermal syndrome-encephalocraniocutaneous lipomatosis (OES-ECCL) and LNSS was assessed in a case series investigation. Large masses involving the cornea and conjunctiva with ill-defined borders were noticed as a distinctive feature of a particular form of epibulbar choristomas. During the research, 26 out of 38 eyes had more than two quadrants of bulbar conjunctiva filled in, and all of them covered the supratemporal quadrant. The parents reported that all masses were not increasing in size. The most common related ocular anomaly, eyelid coloboma, is characterized by the prominent location of the upper inner third of the eyelid. A common occurrence in patients is periocular skin tags, which are found at the site of eyelid colobomas. Fundus anomalies exhibit significant clinical heterogeneity, making it difficult to characterize them in a predictable way [7]. Nonetheless, the most common deformity affecting the optic disc is coloboma [8,9]. The majority of cases had optic disc hypoplasia, which usually manifested as a whitish, decentered excavation of the optic disc or as marginal hypoplasia with surrounding retinal-choroidal atrophy. Vision ranging from 20/200 to finger counting can be associated with congenital disc conformation abnormalities [10]. Therefore, fundus evaluations must be prioritized for patients' routine screening at the earliest opportunity.

Radiographic abnormalities are present in more than 50% of patients with LNSS, with the majority affecting the brain and skull. There have been reports of hamartomas, cortical dysplasia, unilateral ventriculomegaly, and hemimegalencephaly, among the several brain imaging abnormalities associated with LNSS. The most prevalent extracutaneous manifestation of LNSS is neurological involvement. The most common neurological involvements are epilepsy, structural abnormalities, and developmental delays. In the case series research, mosaicism of *KRAS* c.35C>T; p.G12D was discovered in the DNA from the cutaneous lesion of patient 3. A patient with rhabdomyosarcoma also had the mutation found in this study, supporting the notion that there is a growing genetic overlap between cancer and mosaic developmental abnormalities [11-13]. Although the timing of resection for nevus sebaceous therapy is debatable, the majority of experts believe that surgical excision is the best course of action. However, it results in a linear scar. Other treatment options, such as CO_2_ laser treatment, are also available to reduce scarring. However, the lesion might not be completely eradicated by CO_2_ laser vaporization; only the part of the nevus sebaceous located at the epidermis or papillary dermis can be eliminated [14]. Therefore, following laser treatment, there remains a chance of recurrence. Isotretinoin inhibits the production of sebum by reducing the size and metabolic activity of distinct sebocytes, as well as by substantially decreasing the number of sebaceous glands [15]. Therefore, CO_2_ laser treatment in conjunction with isotretinoin may be a suitable alternative. Dermatologists should be aware of the possibility of cancer in any persistent lesions as well as the inadequate eradication of sebaceous nevus. For these patients, periodic and ongoing follow-up is therefore necessary.

## Conclusions

Early referral to an ophthalmologist is essential for patients whose diagnosis is only suspected. The incidence of eyelid coloboma is high in these two conditions. It has been demonstrated that early surgical treatment of eyelid colobomas helps preserve visual development, in addition to enhancing corneal transparency and ocular surface homeostasis. The cooperation of multiple medical specialties is necessary for prompt treatment. Understanding the signs of eye problems can help pediatricians refer patients on time and improve their quality of life by protecting the patient's visual development and appearance. This lessens the strain on the child's family. It is beneficial for patients as well as doctors to be up-to-date on research regarding the clinical characteristics and experiences of these uncommon congenital neurocutaneous diseases.
